# Transcriptional Landscapes of *Herelleviridae* Bacteriophages and *Staphylococcus aureus* during Phage Infection: An Overview

**DOI:** 10.3390/v15071427

**Published:** 2023-06-23

**Authors:** Maria Kornienko, Dmitry Bespiatykh, Roman Gorodnichev, Narina Abdraimova, Egor Shitikov

**Affiliations:** Lopukhin Federal Research and Clinical Center of Physical-Chemical Medicine of Federal Medical Biological Agency Medicine, Moscow 119435, Russia; d.bespiatykh@gmail.com (D.B.); gorodnichev.r.b@gmail.com (R.G.); abdraimovanarina@gmail.com (N.A.); eshitikov@mail.ru (E.S.)

**Keywords:** phage therapy, *Staphylococcus aureus*, *Herelleviridae*, phage–host interaction, total RNA sequencing, transcriptome analysis, RNA-seq

## Abstract

The issue of antibiotic resistance in healthcare worldwide has led to a pressing need to explore and develop alternative approaches to combat infectious diseases. Among these methods, phage therapy has emerged as a potential solution to tackle this growing challenge. Virulent phages of the *Herelleviridae* family, known for their ability to cause lysis of *Staphylococcus aureus*, a clinically significant pathogen frequently associated with multidrug resistance, have proven to be one of the most effective viruses utilized in phage therapy. In order to utilize phages for therapeutic purposes effectively, a thorough investigation into their physiology and mechanisms of action on infected cells is essential. The use of omics technologies, particularly total RNA sequencing, is a promising approach for analyzing the interaction between phages and their hosts, allowing for the assessment of both the behavior of the phage during infection and the cell’s response. This review aims to provide a comprehensive overview of the physiology of the *Herelleviridae* family, utilizing existing analyses of their total phage transcriptomes. Additionally, it sheds light on the changes that occur in the metabolism of *S. aureus* when infected with virulent bacteriophages, contributing to a deeper understanding of the phage–host interaction.

## 1. Introduction

Bacteriophages, also known as phages, have been studied for over a century as viruses that specifically infect bacterial cells. Today, they are widely acknowledged as the most prevalent biological entities on Earth [1,2]. Due to the persistent rise in the global healthcare crisis, marked by the emergence and spread of drug-resistant isolates, the use of phages in the therapy of bacterial infections is becoming a promising alternative to conventional antimicrobial drugs. Phage therapy offers distinct advantages, as phages possess the capability to efficiently lyse both antibiotic-sensitive and antibiotic-resistant bacteria. Moreover, phages demonstrate effectiveness in eliminating biofilms, and their high specificity ensures minimal disruption to the human microbiota, thereby reducing potential negative consequences. Moreover, the process of isolating new phages is straightforward and cost-effective when compared to the development of new antibiotics [3].

The treatment of infectious diseases caused by *Staphylococcus aureus* has shown the most promising results in the field of phage therapy [4,5,6]. *S. aureus* is an opportunistic microorganism that causes diseases of varying severity, such as soft tissue infections, osteomyelitis, pneumonia, infectious endocarditis, meningitis, bacteremia, and others [7]. The mortality rate from staphylococcal infections varies from 5 to 60% depending on the patient population and the location of the lesion [8,9,10]. The difficulty in treating these bacteria with classical antimicrobial therapy arises from their high level of resistance to antibiotics, coupled with their capability of producing a broad spectrum of toxins, such as enterotoxins, leukocidins, and hemolysins. Furthermore, the ability of these pathogens to form biofilms exacerbates the situation [7]. Examples of the successful implementation of virulent phages in combatting infections caused by *S. aureus* have been well documented, particularly in cases of chronic knee prosthetic joint infections [11,12,13], infectious endocarditis, implant-associated cardiac infections [14], wound infections [15], and osteomyelitis [16]. Cases of the successful intravenous use of virulent *S. aureus* phages (staphylophages) as an adjunct to standard therapy have been reported for patients with severe staphylococcal infections, including endocarditis and septic shock [6].

Despite the increasing number of studies highlighting the potential of virulent phages as therapeutic agents, phage therapy is still in its early stages. It is now clear that compared to antibiotic therapy, phage therapy requires consideration of novel factors, including multiplicity of infection, the size and accessibility of the target bacterial population, and the rate of bacterial resistance (evolution) in response to phage pressure. Additionally, the limited comprehension of the molecular mechanisms underlying phage physiology during interactions with bacterial cells is a crucial aspect that requires attention and investigation. The use of transcriptomic analysis can be beneficial for addressing this issue by investigating gene expression within the phage–bacteria system, which is crucial for comprehending gene function during specific infection stages. Transcriptomic analysis involves capturing the complete set of RNA transcripts (transcriptome) in the studied organism. It encompasses assessing the activity of promoters and terminators, identifying the boundaries of transcriptional units, and demonstrating gene expression at different stages of infection. This comprehensive analysis provides a deeper understanding of the dynamics of gene activity throughout the infection process [17]. Various technologies have been developed to study and determine transcriptomes, which include DNA hybridization, DNA microarrays, complementary DNA Amplified Fragment Length Polymorphism (cDNA-AFLP), Expressed Sequence Tags (ESTs) sequencing, Serial Analysis of Gene Expression (SAGE), Massively Parallel Signature Sequencing (MPSS), and RNA sequencing (RNA-seq) [18]. It is important to highlight that RNA-seq, utilizing next-generation sequencing platforms, is widely regarded as the optimal solution in modern bioanalysis, offering virtually limitless possibilities [19].

While the significance of transcriptomic approaches in studying the interaction between virulent phages and their hosts is evident, it is worth noting that the number of studies dedicated to this topic is remarkably limited. Early publications primarily focused on gene expression analysis in order to investigate the interactions between different phages and their respective hosts. These included phages such as *Pseudomonas aeruginosa* (ΦKZ, PaP1, and PAK_P3), *Yersinia enterocolitica* (vb_YecM_ΦR1-37), *Bacillus subtilis* (Φ29), *Campylobacter jejuni* (phage NCTC12673), *Escherichia coli* (phage ΦX174), and *Acinetobacter baumannii* (ΦAbp1) [20,21,22,23,24,25,26,27,28]. Regarding virulent *Staphylococcus* phages, it is worth mentioning three significant studies published in 2022 that focused on transcriptome analysis of phages belonging to the *Herelleviridae* family. These studies include the analysis of phage K [29], as well as the transcriptomic investigations of phages vB_SauM-515A1 and SAM1 [30,31]. This review aims to provide a comprehensive overview of the findings derived from the analysis of the total transcriptomes of the *Herelleviridae* family phages. It aims to provide comprehensive insights into the physiology of these phages, while also exploring the metabolic changes that occur within bacterial cells upon infection with virulent staphylophages.

## 2. Virulent *Staphylococcus aureus* Phages

**Figure 1:**
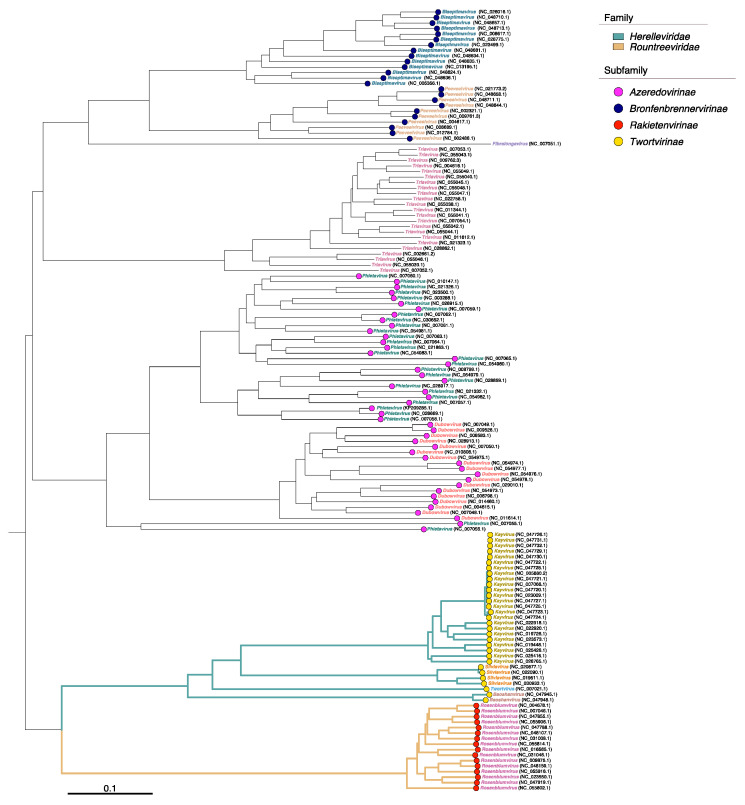
Proteomic tree inferred using 143 ICTV-recommended *S. aureus* phages, reflecting their taxonomic affiliation. The tree was constructed using ViPTreeGen v1.1.3 [32]. The resulting tree was visualized using ggtree v3.8.0 [33] and ggtreeExtra v1.10.0 [34] R packages.

According to the nomenclature of the International Committee on Taxonomy of Viruses (ICTV), all currently known *S. aureus* phages belong to the order *Caudovirales* and are classified into 11 genera. Among these, four genera are classified under the *Herelleviridae* family (formerly a part of *Myoviridae*), while the *Rosenblumvirus* genus is classified under the *Rountreeviridae* family (formerly a part of *Podoviridae*). The remaining genera were previously classified under the *Siphoviridae* family (Figure 1).

The classification of phages as living antimicrobial agents entails specific considerations for their therapeutic application. Notably, for treatment purposes, only virulent phages are utilized, as their replication invariably results in the lysis of bacterial cells [35,36]. Virulent phages are distinct from temperate phages, as they are unable to integrate into the genome of the infected bacterium. This means they do not contribute to the horizontal transfer of genes, which includes determinants of resistance to antibiotics and toxins. The nature of the interaction between staphylophages and bacteria is strictly correlated with taxonomic affiliation. In the case of virulent *S. aureus* phages, they are only found in the families *Herelleviridae* and *Rountreeviridae* [37,38,39]. The *Herelleviridae* family of phages is widely used for therapeutic purposes [40,41,42], primarily for their ability to effectively lyse a significant number of clinical *S. aureus* strains, including those with multiple drug resistance (up to 90%) [40,43]. These phages are also commonly found in natural sources [44]. In fact, the majority of commercial preparations for therapeutic purposes are derived from phages belonging to the *Herelleviridae* family [39,40].

Morphologically, phages of the *Herelleviridae* family are characterized by an icosahedral capsid (diameter about 90 nm) and a long contractile tail (170–240 nm) ending in a basal plate [45,46]. The phage genomes have high similarity (88.3–99.9% identity) and are represented by linear double-stranded DNA of about 127–145 kbp with direct terminal repeats [45]. The genome encodes about 200–250 open reading frames and several tRNA genes. The arrangement of genes in the genome follows a functional module-based organization, which includes morphogenesis, cell lysis, nucleic acid metabolism, and the conversion of host metabolism [45]. The review by Łobocka et al. provides an in-depth examination of the morphological characteristics and comparative genomics of the prevalent phages within the *Herelleviridae* family [45].

**Figure 2:**
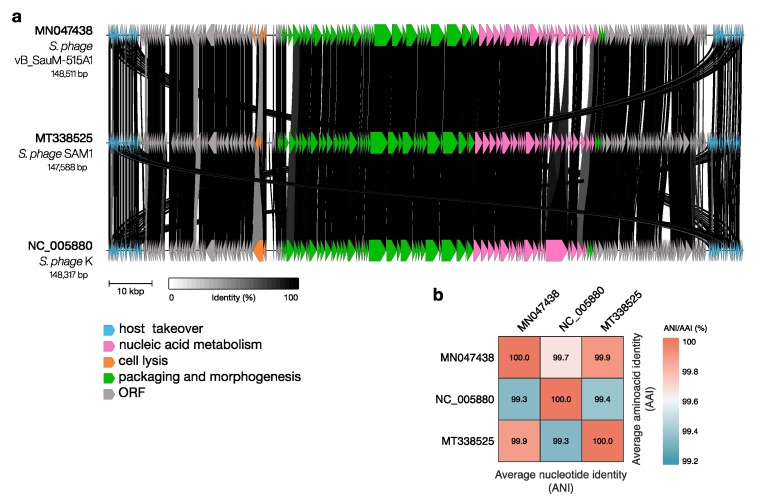
Comparison of genomes of typical phages of the *Herelleviridae* family (vB_SauM-515A1, SAM1, phage K). (**a**) The gene synteny of the three *Herelleviridae* phages. Different colors indicate functional modules. (**b**) The chart displays the average nucleotide identity (lower triangle) and the average amino acid identity (upper triangle) among the three *Herelleviridae* phages. The gene synteny was visualized using clinker v0.0.25 tool [49]; AAI was calculated using EzAAI v1.2.2 [50] and ANI was calculated using FastANI v1.33 [51], visualized using ComplexHeatmap v2.16.0 [52] R library.

The phages that were included in the transcriptional studies also belong to the *Herelleviridae* family and exhibit a high level of genetic similarity (Figure 2). One of the most extensively studied phages in the *Herelleviridae* family is phage K [47]. While the precise origins of phage K remain unclear, it was first described in 1949 [48]. Phage SAM1 was isolated from the preparation Fersisi (named after the G. Eliava Institute of Bacteriophages, Microbiology and Virology, T’bilisi, Georgia) [31]. Phage vB_SauM-515A1 was isolated from the therapeutic preparation “Staphylococcal phage” by NPO “Microgen” and has been described in detail in our previous study [40].

## 3. Features of the Transcriptomics Experiment for the Phage—Bacteria System

Since bacteriophages cannot replicate outside bacterial cells, studying changes in gene expression requires analyzing the transcriptomes obtained from bacterial cultures infected with phages at a multiplicity of infection (MOI) higher than 1 (typically MOI = 10 is used) [28,31,53]. Using an MOI of at least 10 ensures that all bacterial cells within the sample are simultaneously infected with the phage, enabling the analysis of the expression of phage genes as well as the transcriptional response of the infected bacterium. To perform differential gene expression analysis, RNA sequencing data obtained from an uninfected bacterial culture are utilized as a control.

For a more comprehensive understanding of gene expression during the entire infection process, it is advisable to perform RNA sequencing at multiple time points throughout the infection. This provides a more extensive and detailed characterization of gene expression in both the phage and bacteria involved in the infection. The selection of suitable time points for RNA sequencing is guided by the single-cycle growth curve of the phage on the host strain. This curve helps determine the optimal time points for sampling to ensure comprehensive coverage of gene expression throughout the infection process. Typically, the stages of adsorption, latent period, lysis, and stable period are distinguished during the infection. During the adsorption stage, the phage recognizes and binds to the phage-specific receptors on the surface of a bacterium, facilitating the entry of the phage DNA into the cell. During the latent period, the phage genes are actively expressed, modifying the host cell’s metabolism to meet the phage’s requirements. This period involves DNA replication, the assembly of phage particles, and protein synthesis, including the production of proteins responsible for cell lysis (holin, endolysin). Upon reaching the critical concentration of lysing protein, the infected cell undergoes lysis, resulting in the mass release of the phage offspring (also known as the lysis period). After the death of all infected cells, the phage titer does not undergo significant changes (the stable period). Thus, the latent period is the most active stage of phage development and is suitable for transcriptional studies. Phages belonging to the *Herelleviridae* family typically have an average latent period ranging from 30 to 50 min. As a result, samples for transcriptomics analysis are selected at various time points during this period, depending on the specific phage and host strain involved (Table 1).

During the early stages of infection, total RNA-seq analysis typically reveals that the vast majority of the sequenced reads map on to the bacterial genome (>95%). However, as the infection progresses, the phage component rapidly increases, eventually reaching values of 56–72% [29,30]. An increase in the number of reads that map to the phage genome indicates that the cell’s transcriptional apparatus has successfully reorganized for the needs of the phage, and that active production of mRNA has occurred. This pattern has been demonstrated not only for transcriptional studies of virulent staphylophages of the *Herelleviridae* family, but also in experiments with bacteria of other species (such as *Acinetobacter baumannii* and *Bacillus subtilis*) infected with corresponding phages [23,28]. However, it should be noted that in the study by Arroyo-Moreno et al. [31], the number of reads that map to the phage remained almost unchanged during the experiment and was about 50%. In our opinion, the obtained ratio is influenced by the choice of time points for analysis (the first time point is 15 min). Possibly by this time, the amount of phage mRNA in the cell has already significantly increased compared to the initial stages of infection.

## 4. *Herelleviridae* Phage Gene Transcriptional Analysis

### 4.1. Analysis of Phage Promoters and Terminators

As noted previously, RNA sequencing allows determination of the transcriptional landscape of a phage, namely: defining the boundaries of transcriptional units, identifying promoter and terminator regions, and evaluating gene expression at different infection stages. A key aspect of the regulation of gene transcription in a *Herelleviridae* family staphylophage is the absence of its own RNA polymerase. Therefore, all stages of transcription are carried out by cellular RNA polymerase, and gene expression regulation occurs through the modification of the latter using alternative σ factors [45].

The promoters of *Herelleviridae* phages were first annotated through in silico analyses using the complete genome sequences of K, ISP, vB_SauM-fRuSau02, and 812 phages [41,54,55,56]. It should be noted that despite the high degree of homology among the investigated phage genes, the number, structure, and location of promoters significantly differed, which may be related to the use of software designed primarily for analyzing bacterial rather than viral sequences.

**Figure 3:**
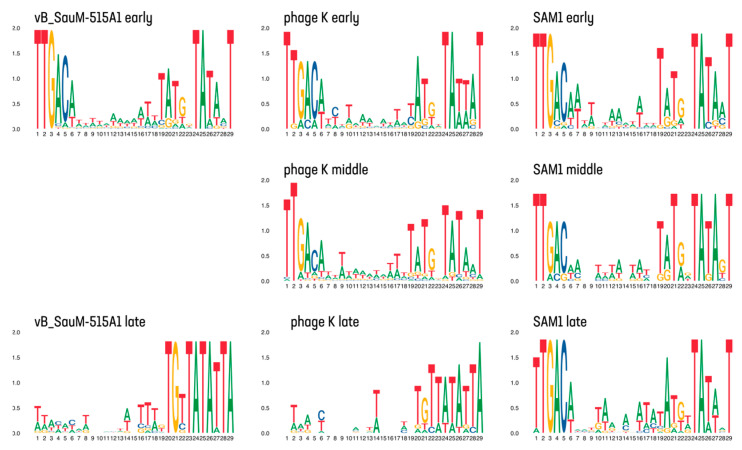
Consensus sequences of different types of promoters of bacteriophages of *S. aureus*. Visualized using ggseqlogo v0.1 [57] and cowplot v1.1.1 [58] R libraries.

The search for promoters by total RNA sequencing allowed the identification of their structure; however, their location, and most importantly, their division into early, middle, and late promoters differed between publications and requires further research. For example, the authors identified early, middle, and late promoters in the analysis of K and SAM1 phages, while only early and late promoters were observed in the analysis of VB_SauM-515A1 (Figure 3).

Notably, the early and middle promoters identified by Finstrlová et al. [29] exhibited a high degree of similarity to each other as well as to the early promoters identified in our own study [30]. One possible explanation for the identification of early promoters could be that the authors incorporated an additional time point (2 min) for their analysis. However, the transition from early to middle promoters is not explicitly addressed in their discussion, nor is the fact that early and middle genes within the same operon are regulated by the same type of promoters. On the other hand, in the study by Arroyo-Moreno et al. [31], there were no significant differences in the early, middle, and late promoters. This observation is consistent with the aforementioned point with regards to the choice of experimental time points, which may have influenced the findings.

The analysis of early and middle promoters of *Herelleviridae* family staphylophages revealed that they belong to the σ70 family of promoters [29,30], which are typical for bacteria, including *S. aureus* [59]. A typical phage promoter of this type has a consensus structure, consisting of a −10 block (TATANT) and an extension element (TRTGN) in front of it, and a −35 block (TTGACW) (Figure 3). A 17 bp spacer is located between the −10 and −35 blocks, which is also a feature typical of σ70 promoters [59]. Spacers of atypical length (16 and 18 bp) were found for some promoters of early phage genes [29,30].

Promoters of late phage genes contain a conservative −10 motif (TGTTATATTA), which is different from that of early and middle genes, and are also characterized by the absence of a −35 motif [29,30]. The putative initiator nucleotide is typically represented by guanine and is situated 6 base pairs downstream of the −10 motif [30].

The RNA sequencing results provided valuable insights into the structure of transcription terminators for both vB_SauM-515A1 (n = 43) and K phages (n = 51) [29,30]. The transcription terminators exhibited a typical structure, which is characteristic of ρ-independent terminators. They featured a loop formed by a GC-rich sequence, followed by a distinctive polyT region [60].

### 4.2. Transcriptional Characteristics of Herelleviridae Phages

**Figure 4:**
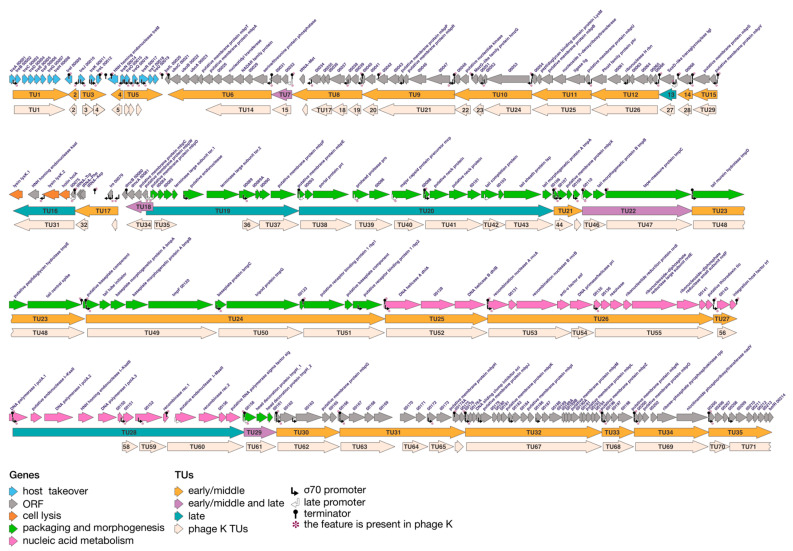
Genome structure and transcriptional landscape of the bacteriophage vB_SauM-515A1 taking into account the analysis data of phage K. Visualized using Gviz v1.44.0 [61] R library and Adobe Illustrator v26.0.2.

By utilizing promoter and terminator location data, it is now possible to predict the types and positions of transcriptional units in a phage. Such an analysis has been conducted only in the studies of vB_SauM-515A1 and K phages [29,30] (Figure 4). However, the same analysis has not been conducted for the phage SAM1 due to the lack of data on terminators.

During the annotation process of the phage vB_SauM-515A1’s transcriptional units, our approach involved considering a transcriptional unit to be bounded by a pair of promoter and terminator elements. It was also acknowledged that a single transcriptional unit could encompass multiple promoters within its boundaries. In contrast, a higher number of transcriptional units were identified for phage K due to a different annotation principle. In the study of phage K, each promoter was considered as the start of a new transcriptional unit. Despite these differences, it is important to emphasize that the boundaries of the transcriptional units for both phages largely coincide.

RNA sequencing analysis revealed that over 80% of the genes in the *Herelleviridae* phages examined are regulated by σ70 promoters (namely, early and middle promoters) and initiate transcription at the early stages of the infection.

Initially, the genes responsible for manipulating the phage genome within the bacterium and facilitating the formation of the phage replicative complex are expressed [29]. Subsequently, there is an active expression of genes associated with the host takeover metabolism, nucleic acid metabolism, and certain genes involved in packaging and morphogenesis. Within the genes responsible for nucleic acid metabolism, it is notable to mention the *sci* gene, as its product exhibits specific inhibition of *S. aureus* DNA replication [62]. The products encoded by morphogenesis genes include various components such as the basal plate, tail fibers, tripod protein, central tail spike, and receptor binding proteins. Additionally, tRNAs are transcribed during the mid-infection phase.

Transcription of early genes does not stop at later stages of infection [30]. This phenomenon arises from the fact that the phage anti-σ factors do not entirely inhibit transcription from strong promoters, such as σ70. Previous studies have demonstrated that the anti-σ factor interacts with the RNA polymerase holoenzyme by binding to the σ subunit. However, this binding selectively affects specific strong promoters that possess a particular sequence within their structure [63,64]. The presumed function of the anti-σ factor is to free a portion of the RNA polymerase for the expression of late genes, rather than completely halting the transcription of the majority of phage genes regulated by σ70 family promoters [30].

During the later stages of the infection process, specific genes are transcribed. These genes encompass structural genes as well as genes responsible for producing products involved in phage particle packaging. The structural genes include the major capsid protein, portal protein, head protease, connector protein, tail sheath protein, protein determining tail length, tail chaperone, tail major protein, and a number of other proteins whose functions are undefined. Additionally, genes that produce hydrolases and the large subunit of terminase, which are involved in phage particle packaging, are also transcribed during this stage. A study conducted by Finstrlová et al. found that the last genes expressed during the infection process are those of holin and endolysin [29].

It is worth noting that in certain cases, late transcriptional units may also be controlled by σ70 promoters. This often occurs when the sequence of the late gene is interrupted by type I intron. Type I introns have been identified in different genes of phages belonging to the *Herelleviridae* family: lysine (*lysK*), DNA polymerase (*polA*), terminase (*ter*), recombinase (*rec*), tail protein (*tmpA*), and ribonucleotide reductase (*nrdI, nrdE*) [45]. Type I introns are represented by the homing endonuclease gene, which is regulated by the early promoter [30,65]. The role of introns in the metabolism of phages belonging to the *Herelleviridae* family remains largely unexplored and requires further investigation.

In addition to the expressed genes within the genomes of the studied phages, the presence of both long non-coding RNAs (lncRNAs) and short non-coding RNAs (sncRNAs) has been identified. However, determining the functions of these non-coding RNAs remains challenging [22,66]. In the case of phages K and vB_SauM-515A1, two similar long non-coding RNAs were expressed at substantial levels. These RNAs exhibited similarities in terms of their position relative to tRNA genes and their transcription levels. Interestingly, these RNAs exhibit similarities in terms of their sequences to the ribozymes GOLLD and ROOL found in *Lactobacillus* prophages [67,68]. It is postulated that the potential role of lncRNAs may be linked to the survival of bacteria under stressful conditions, as exemplified by the RNA OLE in extremophilic bacteria [69]. Additionally, lncRNAs may play a role in regulating the copy number of the phage genome, as observed in the case of lncRNA expression from the megaplasmid of *Lactobacillus salivarius* [68]. Conversely, only phage K has been investigated in terms of sncRNAs and their potential interactions with bacterial targets. It is speculated that these sncRNA sequences might impact the differential expression level of corresponding bacterial genes [29].

## 5. Transcriptional Response of *Staphylococcus aureus*

The RNA sequencing results obtained from the transcriptional experiment provide an opportunity to analyze the impact of the phage on the bacterial host. However, when it comes to the described phage–bacterium pairs, comparing their transcriptional profiles poses several challenges. For instance, studies that examined the transcriptional response of *S. aureus* to the influence of virulent phages from the *Herelleviridae* family were conducted using diverse strains. These strains included clinical isolates and laboratory strains, including one strain devoid of prophages in its genome (*S. aureus* SH1000) (Table 2). Furthermore, the utilization of different growth mediums in the studies contributes to discrepancies in the acquired transcriptional profiles. Additionally, it is important to acknowledge the substantial impact of bioinformatics processing on the observed variations in transcriptional data. In studies investigating the interaction between the clinical isolate *S. aureus* SA515 and phage vB_SauM-515A1 [53], as well as strains *S. aureus* Newman and SH1000 with phage K [29], the analysis of differentially expressed genes (DEGs) was conducted by comparing the infected cultures to an uninfected culture used as a control. In contrast, in the study that examined the impact of phage SAM1 on the clinical strain E1185(IV)ST12 [31], changes in gene expression were assessed relative to the initial time point (15 min). Furthermore, the total number of DEGs varied depending on the selected analysis parameters, such as fold change and *p*-value. Consequently, the percentage of genes showing altered expression in response to the phage ranged from 5.58% to 30.65% of the total number of protein-coding sequences, depending on the specific study (Table 2).

As mentioned above, total RNA sequencing data were obtained for bacterial cultures infected with phage at a high MOI value. Previous studies have shown that at high MOI values, non-specific lysis of the bacterial cell is observed, often mediated by total suppression of macromolecular synthesis and subsequent cell death [70]. Therefore, it is important to note that the bacterial cell response observed in the total RNA sequencing data may not solely result from productive phage infection but could also be influenced by an abundance of phage particles. To gain a clearer understanding, further research is required to elucidate this aspect.

However, it is worth noting that the overall transcriptional response of *S. aureus* during infection with *Herelleviridae* phages exhibits similarities to previously reported transcriptional interactions between phages and their host bacteria [21,22,25,71]. Hence, regardless of the bacterial and phage strains, phage infection induces modifications in the gene expression related to nucleotide biosynthesis, amino acid metabolism, stress response, and nitrogen metabolism [29,53].

In addition to changes in basic metabolic processes, an increase in the expression level of a number of virulence factor genes was shown for the studied *S. aureus* strains (Table 3). The main changes were detected at late stages of infection, and it should be noted that most DEGs were not associated with prophages.

Another notable common feature observed in studies on the response of *S. aureus* to infection is the upregulation of prophage genes. This phenomenon has also been documented in other bacterial species, such as *Pseudomonas aeruginosa* [66] and *Campylobacter jejuni* [25]. It is worth emphasizing that the induction of prophages from the bacterial genome, as mentioned previously, is a phenomenon observed in response to stress [72].

Nevertheless, transcriptomic data reveal changes in expression levels are observed only for specific *S. aureus* prophages (Table 4). This variation could be attributed to the specific type of prophage involved. For instance, studies have demonstrated the hyperexpression of prophage regions in response to virulent phages in several genera, such as *Biseptimavirus* [29,53], *Dubowvirus* [29], and *Phietavirus* [31]. Furthermore, when classifying prophages based on their integrase typing, hyperexpression was observed in prophages carrying the Sa3 [29,53], Sa5 [29,31], and Sa7 [29] integrase types. Notably, prophages with the Sa7 integrase type, even within different taxonomic units, exhibited distinct behaviors when exposed to virulent phages (as shown in Table 4). Hence, the existing data are currently insufficient to draw conclusions about the specific types of prophages induced by virulent phages in *S. aureus*.

The study conducted by A. Finstrlová et al. [29] presents an intriguing hypothesis that connects the upregulation of prophage genes to their competition with virulent phages. Supporting this notion is the discovery of sncRNA in phage K, which targets a conserved mRNA sequence responsible for encoding the basal plate protein (FibU). The interaction between sncRNA and this mRNA sequence has the capacity to impede protein translation, ultimately hindering phage particle assembly [29].

## 6. Perspectives

To ensure the safe utilization of phages as living antimicrobial agents, it is of utmost importance to have a thorough understanding of the physiological characteristics. Currently, transcriptional analysis provides initial insights into the physiology of virulent *Herelleviridae* phages and their interactions with bacterial cells. However, the data obtained from total RNA sequencing vary significantly. Therefore, further experiments are necessary, specifically focusing on different strains infected with phages. These experiments should involve strains with established sequence types, characterized by their full-genome sequencing profiles of virulence factors. Additionally, a thorough characterization of prophages, including detailed information about the type of integrase, should be included in these studies. By conducting such comprehensive investigations, a more complete understanding of phage physiology and their interactions with bacterial cells can be achieved, leading to enhanced safety and effectiveness in their application. Furthermore, it is essential to address the potential limitations of the conducted experiments, such as the use of high multiplicity of infection, which may introduce artifacts in the transcriptional data, particularly related to non-specific lysis. Therefore, to enhance the analysis of transcriptomes and validate the previously obtained data, it is recommended to perform experiments involving RNA sequencing of single infected cells, providing a more precise and reliable understanding of the phage–host interaction.

Additionally, for a detailed characterization of the process of phage infection of *S. aureus* cells, there is an obvious need to attract alternative methods for identifying new features of the process of interaction between the cell and virulent phages, as well as for verifying the obtained data. Furthermore, to achieve a comprehensive understanding of the phage infection process in *S. aureus* cells, it is crucial to explore alternative methods that may uncover new insights into the interaction between the cell and virulent phages. These methods can also serve to validate the obtained data. A particularly promising approach is the utilization of a systems-level analysis to investigate the physiology of phages and their impact on bacterial cells. This involves employing proteomic and metabolomic analyses to gain deeper insights into the molecular dynamics of phage infection. Proteomic analysis, in particular, has proven valuable in virology for characterizing viral proteins, including those found in *S. aureus* phages [41,73]. Proteomics is used less commonly to evaluate changes in protein levels in a cell during phage infection [74]. It is important to highlight the necessity of conducting these studies since, despite the abundance of data obtained from total RNA sequencing, it remains challenging to fully elucidate the intricate interaction between the bacterium and phage. This challenge arises from the incomplete correlation between observed changes at the mRNA level and the corresponding protein-level alterations. Therefore, additional investigations, such as proteomic and metabolomic analyses, are required to provide a more comprehensive understanding of the dynamic interplay between the phage and bacterial cell. These complementary approaches will shed light on the discrepancies between mRNA and protein expression, allowing for a more accurate and detailed description of the bacterium–phage interaction.

As for metabolomics, the integration of metabolomic and transcriptomic analysis data to study the interaction of phages with their hosts has been demonstrated for phages РаР1 and PAK_P3 of *Pseudomonas aeruginosa* [21,24]. Both studies demonstrate the possibilities of modern metabolomics to detect changes in cells related to phage infection. In addition, the feasibility of using metabolomics methods, including nuclear magnetic resonance, to study the interaction of phages with biofilms, has been demonstrated [75]. *S. aureus* bacteria actively form biofilms, which is why methods for eliminating this pathogen in biofilm-associated infections are being developed, including the use of phages. The use of metabolomics methods to track changes caused by virulent phages in biofilms can identify specific biomarkers involved in controlling the stability of the biofilm.

## 7. Conclusions

Total RNA sequencing forms the foundation for molecular investigations into *Herelleviridae* phage physiology and host response. The analysis of transcriptional data has revealed two distinct types of promoters for bacteriophages: σ70 family promoters for early and middle genes, and late gene promoters. Interaction with bacterial RNA polymerase and precise regulation of gene expression via σ70 promoters play pivotal roles since the phages lack their own RNA polymerase. Early-stage gene expression is predominant, while morphogenesis and cell lysis genes are expressed later. Comparative analysis demonstrates the intricate interaction between *Herelleviridae* phages and their hosts, involving stepwise changes and the control of cell metabolism. The response of *S. aureus* cells to phage exposure is systemic, affecting major metabolic pathways and inducing hyperexpression of certain prophage genes.

In summary, total RNA sequencing of bacterial cells infected with phages provides valuable insights into the mechanisms of phage infection, crucial for safe and rational phage therapy. It helps unravel the blockage and restructuring of macromolecular synthesis in bacterial cells, informing the search for new antibacterial agents. Comprehensive analysis data play a crucial role in determining the necessary specifications for bacterial strains employed in phage production and the purification methods of phage lysates for therapeutic purposes. Additionally, combining this analysis with proteomic and metabolomic studies will deepen our understanding of phage infection processes. This integrated approach will enable a transition from empirical to rational phage therapy, grounded on comprehensive molecular insights into phage–cell interactions.

## Figures and Tables

**Table 1:** Description of the main stages of a single growth cycle and the time points for conducting transcriptional analysis of bacteriophages belonging to the *Herelleviridae* family.

Phage	Duration of Adsorption Period (Adsorption of More than 60% of Phage Particles), Min	Duration of the Latent Period, Min	Time Points for Transcriptional Analysis, Min	References
vB_SauM-515A1	5	30–40	5, 15, 30	[30]
K	2	30–35	2, 5, 10, 20, 30	[29]
SAM1	NA ^1^	50	15, 35, 45	[31]

^1^ NA, not available.

**Table 2:** Characteristics of *S. aureus* strains used in RNA-seq studies.

Strain	Strain Type	MLST Type	Genome Size, Mb (GenBank Number)	CDS	Number of DEG	References
SA515	clinical isolate	ST8	2.66 (JAKRSL000000000)	2658	263 (FC ≥ |2|; FDR < 0.001)	[53]
E1185(IV)ST12	clinical isolate	most similar to ST12	2.78 (CP089586)	2704	829 (*p* < 0.05)	[29]
Newman	laboratory strain	ST8	2.89 (NC_009641.1)	2614	625 (FC ≥ |1.5|; FDR < 0.001)	[31]
SH1000	laboratory strain is a derivative of strain NCTC8325, characterized by the absence of prophage and RsbU repair	ST8	2.68 (JAJAFP000000000)	2684	150 (FC ≥ |1.5|; FDR < 0.001)	

**Table 3:** Hyperexpression of *S. aureus* virulence factor genes in different strains.

Virulence Factor	Gene	References
Toxin		
alpha hemolysin	*hlgy*	[29,31]
gamma hemolysin subunit A	*hlgA*	[29]
gamma hemolysin subunit CB	*hlgB*, *hlgC*	[29,31,53]
superantigen-like protein, exotoxin	*set15*	[53]
Adhesins		
fibrinogen-binding protein	*efb*	[53]
fibronectin-binding protein FnbA	*fnbA*	[31]
fibronectin-binding protein FnbB	*fnbB*	[29]
MSCRAMM family adhesin clumping factor ClfA	*clfA*	[29]
extracellular adherence protein Eap/Map	*eap*	[31]
Immune system evasion		
immunoglobulin-binding protein	*sbi*	[29,31,53]
staphylococcal complement inhibitor SCIN	*scn*	[31,53]
chemotaxis inhibiting protein Chp	*chp*	[29]
Virulence factors associated with secretion		
ESAT-6/WXG100 family secreted protein EsxA/YukE	*esxA*	[53]
protein secretion system EssA	*essA*	[53]

**Table 4:** Characteristics of prophages in *S. aureus* strains used in RNA-seq studies.

Strain	Most Similar Phage	Genus	Integrase Type	Hyperexpression
SA515	phi2958PVL (NC_011344)	*Triavirus*	Sa2	−
	phiNM4 (NC_028864.1)	*Phietavirus*	Sa7	−
	P282 (NC_048634.1)	*Biseptimavirus*	Sa3	+
E1185(IV)ST12	3MRA (NC_028917)	*Phietavirus*	Sa5	+
Newman	phiNM1 (NC_008583.1)	*Dubowvirus*	Sa5	+
	phiNM2 (NC_028913.1)	*Dubowvirus*	Sa7	+
	phiNM3 (NC_008617.1)	*Biseptimavirus*	Sa3	+
	phiNM4 (NC_028864.1)	*Phietavirus*	Sa6	−

## Data Availability

Not applicable.
